# Identification of Macrophage Genotype and Key Biological Pathways in Circulating Angiogenic Cell Transcriptome

**DOI:** 10.1155/2019/9545261

**Published:** 2019-05-02

**Authors:** Bert R. Everaert, Steven J. Van Laere, Robrecht Lembrechts, Vicky Y. Hoymans, Jean-Pierre Timmermans, Christiaan J. Vrints

**Affiliations:** ^1^Laboratory of Cell Biology and Histology, University of Antwerp, Antwerp, Belgium; ^2^Laboratory of Cellular and Molecular Cardiology, Antwerp University Hospital, Edegem, Belgium; ^3^Translational Cancer Research Unit, Oncology Center, St-Augustinus Hospital, Wilrijk, Belgium

## Abstract

**Background:**

Circulating angiogenic cells (CAC) have been identified as important regulators of vascular biology. However, there is still considerable debate about the genotype and function of CAC.

**Methods and Results:**

Data from publicly available gene expression data sets were used to analyse the transcriptome of in vitro cultured CAC (CAC_iv_). Genes and pathways of interest were further evaluated using qPCR comparing CAC_iv_ versus CD14^+^ monocytic cells. The CAC_iv_ transcriptome strongly related to tissue macrophages, and more specifically to regulatory M2c macrophages. The cytokine expression profile of CAC_iv_ was predominantly immune modulatory and resembled the cytokine expression of tumor-associated macrophages (TAM). Pathway analysis revealed previously unrecognized biological processes in CAC_iv_, such as riboflavin metabolism and liver X receptor (LXR)/retinoid X receptor (RXR) and farnesoid X receptor (FXR)/retinoid X receptor (RXR) pathways. Analysis of endothelial-specific genes did not show evidence for endothelial transdifferentiation.

**Conclusions:**

CAC_iv_ are genotypically similar to regulatory M2c macrophages and lack signs of endothelial differentiation.

## 1. Background

Endothelial progenitor cell (EPC) therapy is an appealing strategy for the treatment of cardiovascular diseases. The concept of EPCs dates back to a landmark study published in 1997 by Asahara et al. [1], who isolated a ‘putative progenitor endothelial cell' that could be found within the CD34^+^ mononuclear blood cell fraction. These EPCs were able to differentiate *in vitro* into an endothelial phenotype and induce neovascularization *in vivo*. After more than a decade of vigorous research, during which the early findings of Asahara et al. have been extended, the dogma that postnatal neovascularization relies solely on the proliferation, migration and remodeling of fully differentiated endothelial cells, has been largely revised. Nowadays, neovascularization is considered to be a dynamic process in which local endothelial cell proliferation and circulating progenitor cells join forces to engage in the restoration of tissue perfusion.

However, there is still considerable debate about the phenotype and function of EPCs and much of the uncertainty is caused by a high degree of confusion about the definition of EPCs. First of all, over the years, different culture protocols have emerged, all claiming to produce EPCs from peripheral blood mononuclear cells. Furthermore, a variety of molecular marker combinations have been advocated for the characterization of circulating EPCs. Obviously, the ambiguity that surrounds the term ‘EPC' has not facilitated the understanding and advancement of EPC biology.

In the present article, we have investigated an EPC subtype that has been renamed as circulating angiogenic cell (CAC) [2], early EPC [3] or early pro-angiogenic cell (EPC) [4]. For clarity reasons, we will use the term CAC_iv_ for these *in vitro* cultured blood-derived mononuclear cells. The potential use of these cells to aid in the restoration of impaired neovascularization has been investigated [5]. We used gene expression profiling and transcriptome analysis to identify the CAC_iv_-specific gene signature, to determine the CAC_iv_ cytokine-cytokine receptor fingerprint and to investigate the biological processes that are important in CAC_iv_ biology. This approach is not unprecedented, since, for instance in oncology, genetic profiling has revolutionized tumor characterization and yielded new insights into tumor biology [6]. Using transcriptome analysis, several groups were able to make considerable progress in redefining the relationships between the different culture-derived EPC subtypes and other hematopoietic and mesodermal lineage populations. For instance, EPCs cultured with the culture protocol of Hill et al. [7] could be requalified as T-lymphocytes on the basis of their gene signature [8].

In the present paper, we provide evidence that CAC_iv_ strongly relate to tissue macrophages, and more specifically to regulatory M2 macrophages, without evidence for endothelial transdifferentiation.

The cytokine expression profile is predominantly immune modulatory and resembles the cytokine expression of tumor-associated macrophages (TAMs). Pathway analysis has revealed previously unrecognized biological processes in CAC_iv_, such as riboflavin metabolism and liver X receptor (LXR)/retinoid X receptor (RXR) and farnesoid X receptor (FXR)/retinoid X receptor (RXR) pathways. Together, our findings provide novel insights into the field of CAC biology.

## 2. Methods

### 2.1. Ethics Statement

The data that is reported in this manuscript used publicly available published data sets from other studies. The data collected from GSE2040 involved cell cultures of human volunteers and the data obtained from GSE5099 involved cell material from blood donor buffy coats. Both studies were in compliance with the Helsinki Declaration on research involving human subjects, human material or human data and under the approval of an appropriate local ethics committee. For a qPCR study of CAC_iv_, we collected blood of healthy volunteers. The CAC_iv_ culture protocols were reviewed and approved by the local ethics committee of the Antwerp University Hospital (EC number 12/10/101). Written informed consent was obtained from all participants.

### 2.2. Microarray and Pathway Analysis

To develop the CAC_iv_ gene signature, a publicly available gene expression data set (GSE2040) (HG-U95Av2 microarray (Affymetrix Inc.), see supplementary data file (available here)), targeting 9,670 human genes as selected from the National Center for Biotechnology Information (NCBI) Gene Bank database, was retrieved from the NCBI website (https://www.ncbi.nlm.nih.gov). This gene expression data set contained 3 gene expression profiles of CACiv and 3 gene expression profiles of CD14+ monocytes, all of which were included in the analysis. Raw expression data were normalized using GCRMA and probe sets with a fluorescence intensity above 100 in at least 25% of the arrays were filtered for further analysis. The gene signature was generated using the nearest shrunken centroid method implemented in the R-package Prediction Analysis of Microarrays (PAM). Using a leave-one-out cross-validation procedure, a ∂-value was selected in such a way that the misclassification error rate was minimal. The global clustering pattern of the CAC_iv_ signature genes was evaluated using unsupervised hierarchical clustering (UHC) with the Euclidean distance as distance measure and complete linkage as the dendrogram drawing method. Using the global test [9] we evaluated global differences in expression for probe sets annotated to the KEGG pathway ‘cytokine-cytokine receptor interaction' (map04060) between CAC_iv_ and CD14^+^ monocytes.

The Ingenuity Pathways knowledge base Analysis (IPA) (Ingenuity® Systems, http://www.ingenuity.com) software was used to identify biological networks, functions and canonical pathways important to CAC_iv_ biology. To appreciate the genetic resemblance of the CAC_iv_ gene expression profile in relation to other cell types of interest (i.e., macrophages, monocytes, endothelial cells), the expression of CAC_iv_ signature-related genes was analyzed using the Reference database for gene Expression Analysis (RefExA, http://www.lsbm.org/database), together with an extensive review of the literature.

The following strategy was adopted to evaluate the possible macrophage genotype of cultured CAC_iv_. A microarray data set (GSE5099, HG-U133A (Affymetrix Inc.), see supplementary data file) including a total of 44928 entries representing more than 33,000 human genes containing expression data on the differentiation of monocytes into macrophages, and of macrophages into an M1 or M2 macrophage subtype was downloaded from Gene Expression Omnibus (https://www.ncbi.nlm.nih.gov/geo). Raw expression data were normalized using GCRMA and probe sets with a fluorescence intensity above 100 in at least 25% of the arrays were filtered for further analysis. Using PAM, we generated gene centroids for the monocyte, macrophage, M1 macrophage and M2 macrophage cell fractions. The leave-one-out cross-validation procedure was used to select a ∂-value in such a way that the misclassification error rate was minimal. The centroids were applied onto the GSE2040 data set using the nearest centroid classification routine. Samples were classified by correlating the centroid-specific gene expression profile of each sample in the data set with the shrunken centroids generated by the PAM algorithm. Positive correlation coefficients indicate resemblance of the tested sample to the cell fraction represented by the centroid. Mann–Whitney *U* tests were used to compare the resulting correlation coefficients between CAC_iv_ and monocytes.

To evaluate the degree of resemblance between CAC_iv_ and endothelial cells, we retrieved an endothelial-specific gene list reported by Bhasin et al. [10]. We used UHC analysis (Euclidean distance, complete linkage) to assess global differences between CAC_iv_ and endothelial cells with respect to the endothelial-specific gene list. In addition, we calculated the average gene expression of the endothelial-specific genes and compared this level between CAC_iv_ and CD14^+^ monocytes to evaluate whether a difference in expression existed between both cell types with respect to the set of endothelial-specific genes.

The data that is reported in this manuscript used publicly available published data sets from other studies. The data collected from GSE2040 involved cell cultures of human volunteers and the data obtained from GSE5099 involved cell material from blood donor buffy coats. Both studies were in compliance with the Helsinki Declaration on research involving human subjects, human material or human data and under the approval of an appropriate local ethics committee.

### 2.3. Cell Isolation and Cell Culture

Mononuclear cells were extracted out of blood specimens of healthy volunteers (*n* = 4) by density gradient centrifugation using lymphocyte separation medium (Lonza). The CD14^+^ cell fraction was isolated by using CD14 MicroBeads (Miltenyi Biotec) according to the manufacturer's instructions. CAC_iv_ were cultured out of the mononuclear cell fraction of blood specimens of healthy volunteers (*n* = 4) according the method first described by Dimmeler et al. [4] In brief, 10^6^ mononuclear cells were plated on human fibronectin-coated 24-well culture dishes and maintained in EBM-2 basal medium with EGM-2-MV SingleQuots and 20% fetal bovine serum (FBS) (Lonza). After 3 days in culture, nonadherent cells were removed by washing with PBS and adherent cells were further incubated in fresh medium until day 7. Human umbilical vein endothelial cells (HUVEC) were purchased from Lonza and cultured in EBM-2 basal medium with EGM-2 SingleQuots for 14 days (*n* = 2, technical replicates). Cells from passage 6 and 7 were used.

### 2.4. RNA Extraction and Quality, cDNA Synthesis

RNA was isolated using the RNeasy mini kit (Qiagen) following the manufacturer's instructions. On-column DNAse treatment (Qiagen) was used to remove contaminating DNA leftovers. RNA concentration and purity were analyzed using Nanodrop spectrophotometer (Nanodrop technologies) readings at 260 and 280 nm. Complementary DNA (cDNA) was synthesized using Invitrogen superscript kit according to the manufacturer's instructions and using random hexamer primers for reverse transcription. Reverse transcription was performed at 50°C for 55 minutes, followed by 5 minutes of incubation at 85°C to inactivate the reverse transcriptase enzyme. cDNA samples were placed on ice and stored at -20°C until further use.

### 2.5. qPCR

Taqman® gene expression assays (Applied Biosystems) were used for qPCR analysis on a LightCycler® 480 instrument (Roche). All primers were designed to be intron spanning. qPCR was performed using the LightCycler® Taqman® Master Mix (Roche) in a final reaction volume of 20 *μ*l. We used the geNorm algorithm to determine an optimal combination of reference genes for internal normalization (i.e., *GAPDH* and *HPRT*). All qPCR reactions were carried out as follows: after an initial denaturation-activation step at 95°C for 10 min, amplifications consisted of 45 cycles of denaturation at 95°C for 10s, annealing at 60°C for 15 s and measurement of fluorescence at 72°C for 1 s. Cycle number (Cq) was measured using the baseline-independent second derivative maximum method. Normalized relative gene expression was determined by the E^-∆∆Cq^ method. Assay efficiency (E) was measured by serial dilution of cDNA of pooled samples based on the slope of the standard dilution curve (E = 10^(1/-slope)^-1).

### 2.6. Statistical Analysis

Statistical analysis was performed in PASW® statistics 18 (IBM Corp.). Graphs were created in GraphPad Prizm®. Data are expressed as mean ± SEM. Student's t-test was used for statistical analysis of relative expression data after logarithmic transformation because of non-normality of data subsets. A two-sided *p*-value of <0.05 indicated statistical significance.

## 3. Results

### 3.1. CAC_iv_ Gene Signature Closely Resembles M2 Macrophage Transcriptome, with Little Evidence of Endothelial Cell Transdifferentiation

**Figure 1:**
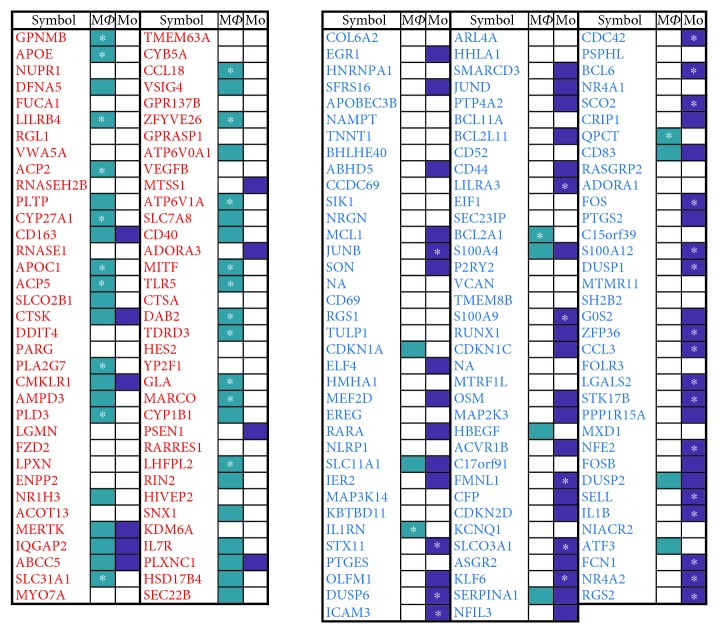
CAC_iv_ gene signature. 70 genes were significantly upregulated versus 107 genes were significantly downregulated in CAC_iv_ versus CD14^+^ monocytic cells. The upregulated (red) and downregulated (blue) genes express a close lineage relationship with macrophages and monocytes, respectively. We highlighted those genes of the CAC_iv_ signature that are specific for either macrophages or monocytes. ^∗^: very specific. M*ϕ*: macrophage; Mo: monocyte.

We composed a gene signature that discriminates between CAC_iv_ and CD14^+^ monocytes using a publicly available gene expression data set (GSE2040) that has previously been used to investigate the neovascularization capacity [11] and production of cytokines by CAC_iv_ [12] and to validate the proteomic characterization of CAC_iv_ [4]. Using the nearest shrunken centroid method, we identified 70 genes that were significantly upregulated versus 107 genes that were significantly downregulated in CAC_iv_ versus CD14^+^ monocytic cells (∂ = 2.85, misclassification error rate = 0) (Figure 1).

**Figure 2:**
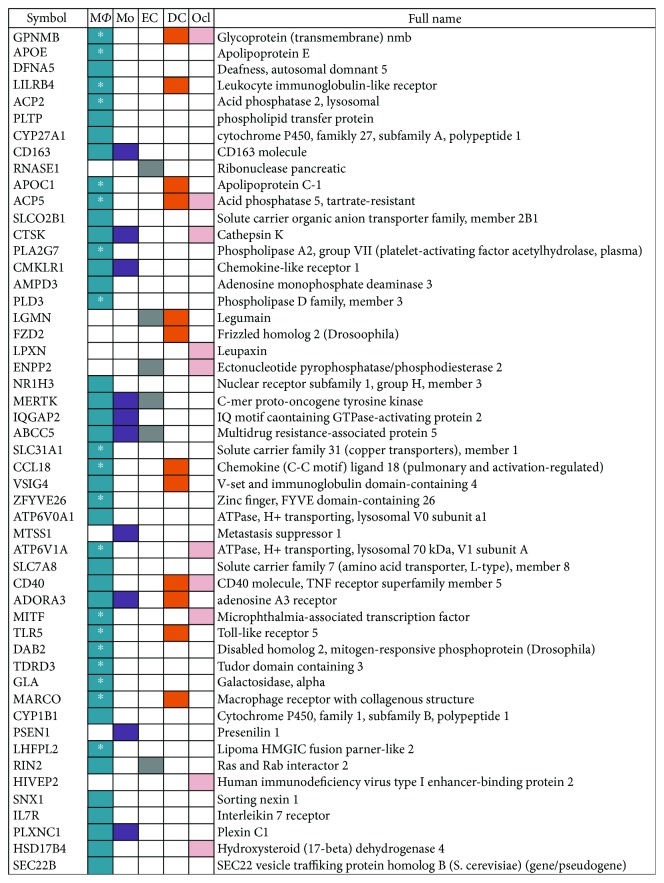
Expression of CAC_iv_ signature related genes in myeloid cells. To demonstrate the lineage relationships between CAC_iv_ and other cell types, we highlighted those genes of the CAC_iv_ signature that were found to be significantly upregulated and also specific for a particular cell type. ^∗^: very specific for macrophages. M*ϕ*: macrophage; Mo: monocyte; EC: endothelial cell; DC: dendritic cell; Ocl: osteoclast.

Comparison of the CAC_iv_ signature with the cell specific expression data in the RefExA database yielded abundant evidence for macrophage-associated gene expression or genes that were related to monocyte/macrophage differentiation, such as GPNMB, APOE, APOC1, ACP2 and CYP27A1. In total, 43 of the 70 CAC_iv_ signature genes showed to be highly expressed and/or specific for macrophages. Considering the downregulated genes, repression of the monocytic lineage transcriptome was evident (e.g. *RGS2*, *NR4A2*, *FCN1*, *IL1B* and *SELL*). Furthermore, the CAC_iv_ signature revealed the upregulation of several genes associated with the differentiation and function of DCs (*CD40*, *MARCO*, *FZD2*, *LILRB4* and *LGMN*) and osteoclasts (*ACP5*, *CTSK*, *LPXN*, *ATP6V1A* and *MITF*) (Figure 2).

**Figure 3:**
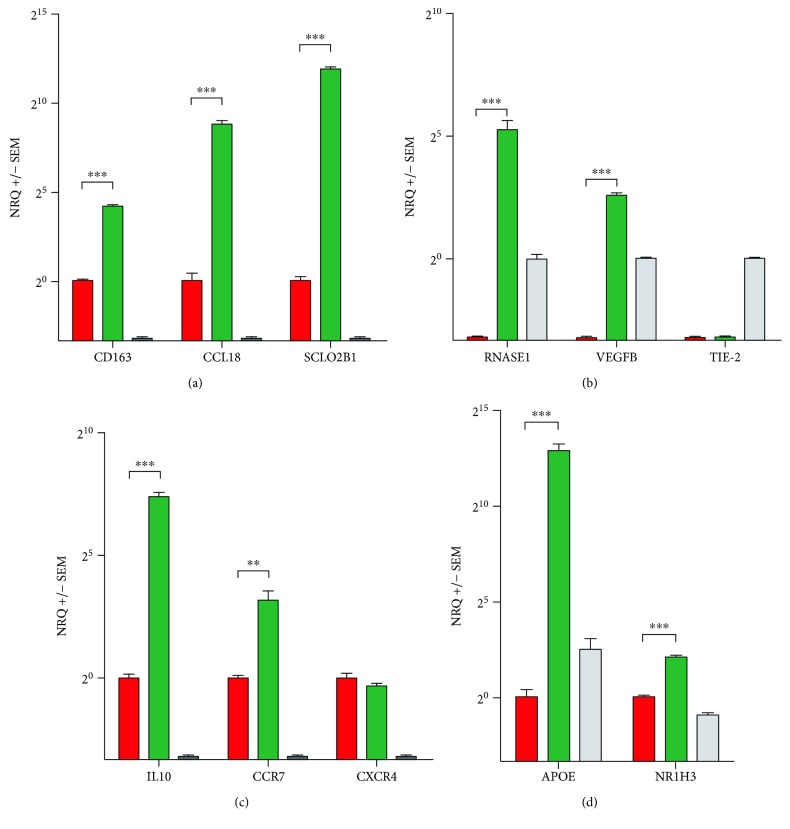
Gene expression analysis. Gene expression of (a) M2c macrophage-related, (b) endothelial, (c) cytokine/cytokine receptor and (d) cholesterol transporter pathway genes in CAC_iv_ (green) vs. CD14^+^ monocytes (red) vs. human umbilical vein endothelial cells (HUVEC) (grey). Only the relevant statistical significance between CACiv and CD14^+^ monocytes is depicted. NRQ: normalized relative quantity. ^∗∗^ *p* < 0.01; ^∗∗∗^ *p* < 0.001.

Interestingly, the expression profile of the CAC_iv_ evidenced a high resemblance to that of ‘alternatively activated' M2 macrophages, and more specifically to the M2c subtype that is induced by the anti-inflammatory cytokine IL10 and is characterized by the upregulation of CD163 and CCL18 [13]. Both *CD163* and *CCL18*, together with other markers specific for M2 differentiation of macrophages, such as *SLCO2B1*, were identified in the CAC gene signature. The upregulation of the M2c trait was further substantiated with qPCR gene expression analysis of *CD163*, *CCL18* and *SCLO2B1* in CAC_iv_ from healthy volunteers compared to CD14^+^ monocytes and HUVEC (Figure 3(a)).

Additionally, the expression of M1 inflammatory macrophage differentiation markers, such as ATF3, IL1B and CCL3, was found to be significantly downregulated in the microarray data. Interestingly, we identified several genes in the CAC_iv_ signature that are known to be implicated in lipid processing (*PLTP*, *NR1H3*) and plaque remodeling by atherosclerotic plaque-associated macrophages (*CTSK*, *LGMN and VSIG4*), and additionally observed repression of several genes regulating transformation of macrophages into foam cells (*RGS2*, *NR4A2 and S100A12*).

**Figure 4:**
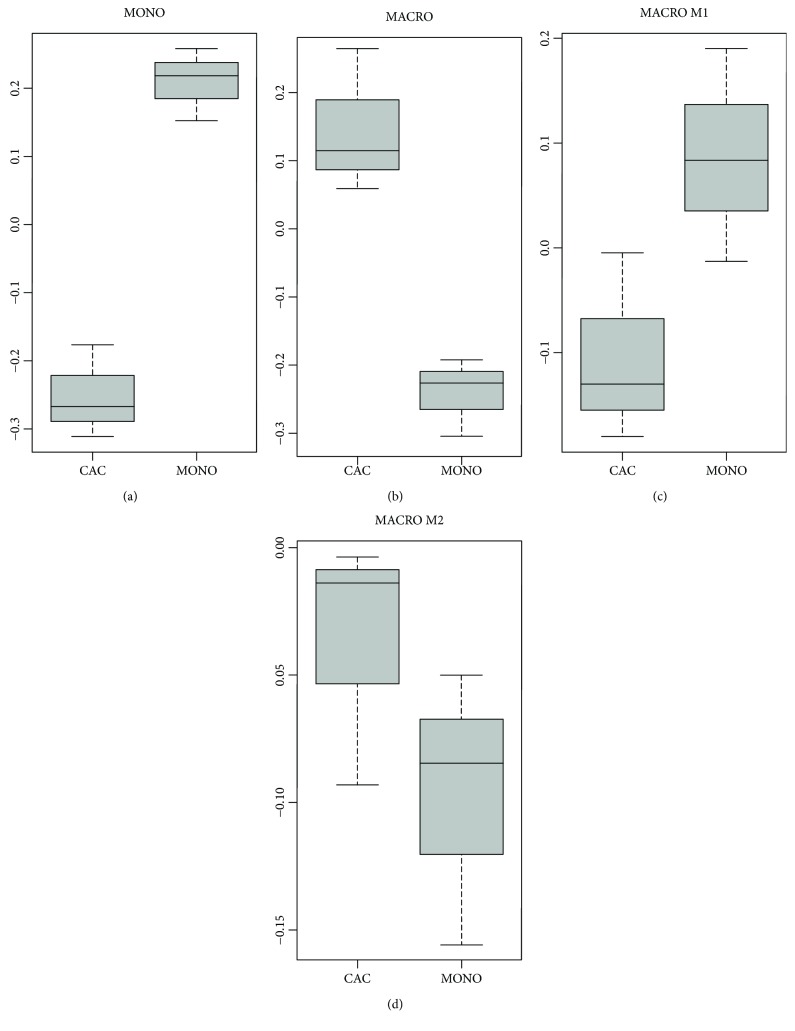
CAC_iv_ differentiate towards a predominanty M2 macrophage genotype. Boxplots comparing the relative expression of the genes in (a) the monocyte-specific gene signature (MONO), (b) the macrophage-specific gene signature (MACRO), (c) the LPS- and IFN-*γ*-stimulated M1 macrophage-specific gene signature (MACRO M1), and (d) the IL4-stimulated M2 macrophage-specific gene signature (MACRO M2), between CAC_iv_ and CD14^+^ monocytic cells (MONO). Positive values signify a stronger degree of similarity of the genetic expression of CAC_iv_ or CD14^+^ monocytic cells to the specified cell type.

To elaborate further on the resemblance of the M2 macrophage genotype and the transcriptome of cultured CAC_iv_, we used a microarray data set (GSE5099) on the differentiation of monocytes into macrophages, and of macrophages into the M1 or M2 macrophage subtype (macrophages cultured for 18 h with LPS and IFN-*γ* or IL4, respectively). We found that the CAC_iv_ expression profile was discordant with the monocytic cell signature (*p* = 0.001) but significantly correlated with the signature of macrophages (*p* = 0.011). Concerning M1 versus M2 differentation, CAC_iv_ exhibited a predominant M2 expression profile (Figure 4).

Surprisingly, however, our CAC_iv_ signature revealed little to no evidence for endothelial cell (EC) differentiation. We compared the CAC_iv_ signature to the RefExA database and two published data sets of endothelial restricted genes [10, 14]. Except for *RNASE1*, we found no evidence for induction of endothelial-specific gene expression in the CAC_iv_. Furthermore, comparison of the CAC_iv_ signature to a published gene signature of tumor-derived endothelium (ovarian carcinoma) yielded only one tumoral vascular marker, *GPNMB*, which was upregulated in both profiles [15]. qPCR analysis of freshly cultured CAC_iv_ from healthy volunteers confirmed *RNASE1* and *VEGFB* upregulation in CAC_iv_ versus CD14^+^ monocytes and HUVEC. However *TIE-2 (TEK)* expression pertained exclusively to HUVEC whereas both CAC_iv_ and CD14^+^ monocytes failed to show any *TIE-2* expression (Figure 3(b)). This finding restricts CAC_iv_ from transdifferentiating into the endothelial cell lineage.

**Figure 5:**
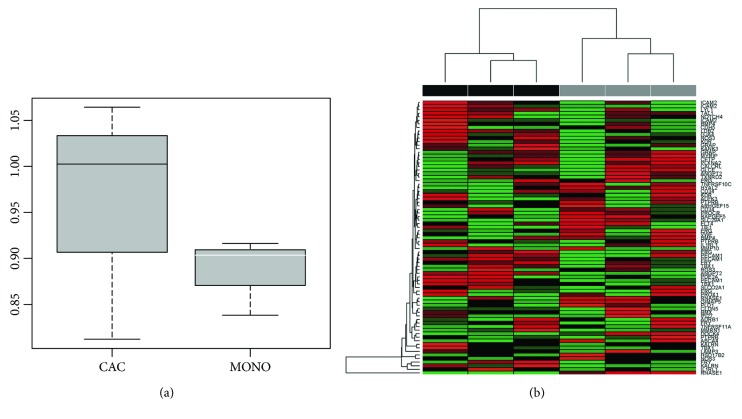
Analysis of EC-associated genes in the CAC_iv_ profile. (a) Boxplot comparing the relative expression of an endothelial gene set between CAC_iv_ and CD14^+^ monocytic cells. There is no significant difference in similarity of the genetic expression of CAC_iv_ or CD14^+^ monocytic cells to an EC-specific gene signature. (b) UHC analysis highlighting the relative expression of EC-associated genes in CAC_iv_ (grey) and monocytes (black). *RNASE1* is indicated as most differentially expressed in CAC_iv_ compared to CD14^+^ monocytic cells. We observed a lack of consistency in EC-associated gene expression between different CAC_iv_ culture samples. Red: upregulation; green: downregulation.

To investigate whether the CAC_iv_ transcriptome showed higher resemblance to endothelial cells than CD14^+^ monocytes, we performed UHC analysis using an endothelial cell-specific gene set (derived from [10]) to cluster CAC_iv_ versus CD14^+^ monocytes. Overall, CAC_iv_ did not demonstrate higher differentiation capacity towards the endothelial profile compared to CD14^+^ monocytes (Figure 5). *RNASE1*, also present in the EC signature, stood out in both the UHC and qPCR analysis as highly upregulated gene. This is interesting and may point to an important function of this gene in CAC biology.

Together, these findings do not seem to support the hypothesis that CAC_iv_ give rise to cells with an endothelial genotype, but would rather suggest that CAC_iv_ closely resemble M2c macrophages.

### 3.2. CAC_iv_ Express a Cytokine Profile Compatible with Regulatory M2 Macrophages and TAM

**Figure 6:**
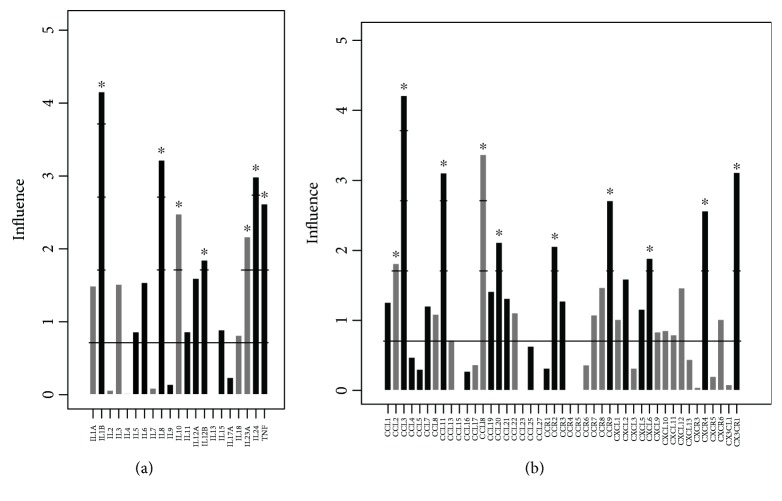
CAC_iv_ -related cytokine and chemokine/chemokine receptor expression profile. Gene expression plot depicting (a) cytokines or (b) chemokines and chemokine receptors that were differentially overexpressed in CAC_iv_ (grey) vs. monocytes (black). The influence (*y*-axis) represents the number of standard deviations (SD) the gene expression of each gene exceeds the null hypothesis that there would be no difference between both groups (z-score). Genes with an influence of ≥1.96 show a statistically significant differential gene expression (*p* < 0.05). ^∗^ *p* < 0.05.

To gain further insight into CAC_iv_ biology, we investigated their cytokine and cytokine receptor expression. Using a global test, we evaluated differences in cytokine-cytokine receptor expression for genes annotated to the KEGG pathway ‘Cytokine-cytokine receptor interaction' and were able to demonstrate clear differences in interleukin, chemokine and chemokine receptor expression between CAC_iv_ and monocytes. Monocytes exhibited a classical inflammatory genotype, with expression of inflammatory cytokines, such as IL1*ß*, (IL6), IL8, (IL12*α*), IL12*ß* and TNF (Figure 6(a)). CAC_iv_, on the other hand, showed significant upregulation of the anti-inflammatory cytokine IL10 and the matrix remodeling, pro-angiogenic cytokine IL23*α*. Increased *IL10* expression combined with reduced expression of inflammatory cytokines, is characteristic of the M2c macrophage subtype, in the literature referred to as regulatory macrophages [16].

CAC_iv_ significantly expressed the chemokines CCL2 and CCL18 (Figure 6(b)). Other chemokines, such as CCL17 and CCL22, showed a trend towards increased expression in CAC_iv_. Interestingly, the chemokine combination of CCL17, CCL18 and CCL22, is a cluster that has been associated specifically with the M2 macrophage profile. However, because *CCL17* and *CCL22* expression did not reach statistical significance, this association in CAC_iv_ remains speculative. Since also the M1 macrophage-associated chemokine CCL2 was upregulated in CAC_iv_, this chemokine profile strongly resembles the chemokinetic fingerprint of TAM [17]. Moreover, TAM are characterized by low expression of inflammatory cytokines, such as IL1*ß*, IL12*ß* and TNF, lending further support to the similar genotypic appearance of CAC_iv_ and TAM.

Interestingly, CAC_iv_ also expressed a number of M1 macrophage-associated pro-inflammatory markers, such as IL1*α* and IL23*α*. Stimulation of cardiac myofibroblasts with IL1*α* has been shown to lead to the production of extracellular matrix metalloproteinases (MMPs), such as MMP2 and MMP9, and inhibits the expression of ADAMTS1, an angiogenesis inhibitor [18]. IL23*α* has also been shown to upregulate MMP9 activity and enhance angiogenesis [19]. Together, the expression of these M1-associated cytokines could endow CAC_iv_ with important tissue remodeling and angiogenic properties in the setting of MI.

Finally, we identified the CCR7 chemokine receptor as the most differentially expressed chemokine receptor in Calthough its expression did not reach statistical significance. This chemokine receptor is commonly found on mature DCs [20] and facilitates lymphoid tissue homing. Interestingly, CXCR4, the receptor for the hypoxia-inducible chemokine CXCL12, showed higher expression on monocytes than on CAC_iv_, suggesting that differentiated CAC_iv_, do not display increased hypoxia-directed tissue homing capacity compared to CD14^+^ monocytic cells. On qPCR analysis, the expression of *IL10* and *CCR7* was comfirmed to be significantly upregulated in CAC_iv_ (Figure 3(c)).

### 3.3. Pathway Analysis

**Figure 7:**
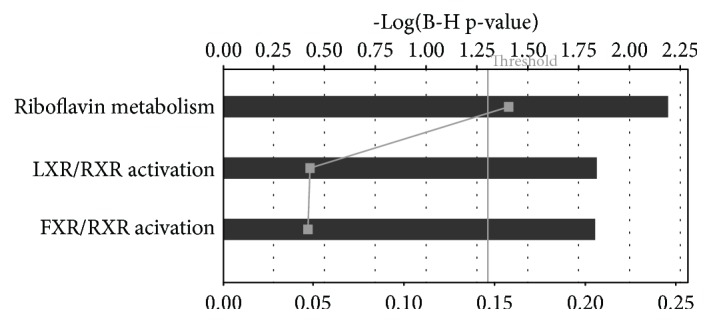
Summary of IPA analysis. The main upregulated canonical pathways are shown. Riboflavin metabolism and LXR/RXR and FXR/RXR activation remain statistically significant even after correction for multiple comparisons (Threshold indicates false discovery rate corrected p-value of 0.05). Bottom axis depicts the ratio of the number of genes upregulated in de CAC_iv_ signature divided by the total number of genes involved in a given pathway (squares).

Using IPA software, we determined the biological networks, functions and canonical pathways important to CAC_iv_ biology. The main CAC_iv_-associated biological networks and molecular functions consisted of genes implicated in lipid metabolism, molecular transport, biochemistry of small molecules, inflammatory responses and cardiovascular disease. LXR/RXR (*APOC1*, *APOE*, *NR1H3*, *PLTP*; *p* = 2.9 × 10^−4^) and FXR/RXR (*APOE*, *CYP27A1*, *NR1H3*, *PLTP*; *p* = 4.46 × 10^−4^) signaling pathways and riboflavin metabolism (*ACP5*, *ACP2*, *ENPP2*; *p* = 6.54 × 10^−5^) were significantly upregulated in CAC_iv_ compared to CD14^+^ monocytes (Figure 7), whereas genes involved in inflammatory pathways and the acute phase response were repressed, highlighting the anti-inflammatory properties of CAC_iv_. Interestingly, a gene cluster consisting of IL10 antagonistic factors (*MAP3K14*, *FOS*, *DUSP1*, *IL1RN*, *CDKN1A*, *IL1B*, *PTGS2*, *CCL3*, *CDKN1C*; *p* = 3.24 × 10^−5^), was found to be associated with the monocyte-like profile and appeared to be repressed during differentiation towards CAC_iv_. This finding is in accordance with the cytokine expression profile of CAC_iv_ and is indicative of a shift towards immune-modulatory IL10 signaling pathways. The upregulation of the FXR/RXR and LXR/RXR associated genes *APOE* and *NR1H3* in CAC_iv_ compared to CD14^+^ monocytes was confirmed by qPCR analysis (Figure 3(d)).

## 4. Discussion

In this article we used an in silico microarray analysis approach to explore the genotype of CAC_iv_ and were able to identify a gene expression profile characteristic of CAC_iv_. The expression of several key genes was further substantiated with qPCR analysis. We compared the CAC_iv_ transcriptome with microarray data sets dealing with monocyte-macrophage and endothelial cell differentiation and found considerable evidence for macrophage lineage differentiation in the CAC_iv_ genotype but, surprisingly, little evidence for endothelial transdifferentiation. Further analysis of the nature of CAC_iv_ showed high correlation of the CAC_iv_ gene signature with the M2 macrophage subtype. Because macrophage M1 and M2 subtypes merely represent the extremes on a wide spectrum of possibilities of macrophage polarization and since we also found some conserved M1 lineage characteristics in the CAC_iv_ signature, CAC_iv_ most probably constitute a specific intermediate macrophage subtype, with predominant traits of regulatory M2c macrophages [16].

Several studies have dealt with the lineage commitment of EPC. A first study questioning the true progenitor cell nature of EPC was published by Rehman et al. [2], who revealed that mononuclear cells cultured for only a short period under EPC culture conditions mainly yielded cells that expressed monocytic markers, such as CD14, CD11b, CD11c and CD168, as such confining them to the monocyte/macrophage lineage. Considering the lack of evidence for endothelial cel differentation, Rehman renamed these cells as CAC. Furuhata et al. [21] compared the characteristics of cultured CD34^+^ mononuclear cells with mature endothelial cells. Using hierarchical clustering, this group reported the absence of endothelial-specific marker expression, such as Tie2, angiopoietin-2, VE-cadherin, endoglin or KDR, even after 14 days in culture and found a high expression of macrophage-specific markers, such as GPNMB, matrix metallopeptidases 7 and 9, lysosomal acid lipase and APOE. Medina et al. [22], revealed a clear distinction in gene signature between CAC_iv_ and (late) outgrowth endothelial cells (OEC), also known as endothelial colony-forming cells (ECFC). ECFCs were closely related to endothelial cells, whereas the CAC_iv_ genotype clustered with monocytic cells and evidenced an alternative activated M2 macrophage genotype [23]. Finally, a UHC analysis study of Gremmels et al. [24] provided data on the relation of CAC_iv_ and ECFC with various other cell types of hematopoietic and mesenchymal origin and concluded that CAC_iv_ display a genotype that is restricted to the hematopoietic lineage, whereas ECFC, together with endothelial cell subtypes, belong to a large mesenchymal cell cluster.

The absence of endothelial markers in the CAC_iv_ signature is evident and, together with the evidence from previous studies, almost excludes direct transdifferentiation of CAC_iv_ into EC. Most likely, as demonstrated by Prokopi et al. [25], this hypothesis of transdifferentiation is the result of assay misinterpretation due to the contamination with platelet-derived microparticles of conventional mononuclear cell isolation procedures.

Macrophages show a remarkable degree of plasticity in response to specific environmental stimuli and many distinct macrophage subsets have been described [26]. Broadly speaking, macrophages are polarized along a spectrum of two extremes, M1 and M2 macrophages, which have different genotypes and function. M1 macrophages produce inflammatory cytokines, such as IL1*ß* and TNF, play a role in Th1 responses and in the killing of pathogens and tumor cells. M2 macrophages, on the other hand, display anti-inflammatory properties, facilitate Th2 responses and engage in active tissue remodeling and tumor promotion. Recently, this dichotomized view on macrophage differentiation was challenged by a new paradigm [16] according to which macrophages are classified based on physiological activities, such as host defence, wound healing and immune regulation. Moreover, macrophage subtypes are thought to reflect ‘blends' of these basic macrophage ‘flavors', resulting in a tissue or disease-specific macrophage genotype. Because we also found expression of markers specific for osteoclasts and DCs, the CAC_iv_ profile probably reflects a macrophage subset with M2 predominance, closely resembling regulatory M2c macrophages. Still, CAC_iv_ express some M1-associated pro-inflammatory markers, such as IL1*α* and IL23*α*, which could aid CAC_iv_ to engage in important biological processes, such as tissue remodeling and angiogenesis. Additional studies, comparing the CAC_iv_ transcriptome with these and other macrophage-related cell types, are warranted.

Using IPA analysis, we identified riboflavin metabolism and the LXR/RXR and FXR/RXR pathways as the most significantly upregulated biological processes in the CAC_iv_ transcriptome.

LXRs are a family of cholesterol-sensing nuclear receptors regulating lipid homeostasis and cholesterol transport [27]. In macrophages, LXRa gene expression has been shown to be upregulated during monocyte to macrophage differentiation [28]. Treatment of ApoE-deficient atherosclerosis-prone mice with LXR agonists significantly reduced atherosclerotic lesion formation [29], highlighting the atheroprotective effects of LXR pathway induction. Interestingly, upregulation of the LXR pathways together with the DC chemokine receptor CCR7 has been reported in CD68^+^CD14^−^ macrophages in normal intima devoid of atherosclerotic disease [30]. The CAC_iv_ expression profile resembled that described by Trogan et al. [31], who showed in laser-capture microdissection-isolated foam cells that induction of the LXR pathway genes combined with increased CCR7 expression was a hallmark of atherosclerotic plaque regression and that atherosclerotic regression could be inhibited by targeting the CCR7 ligands CCL19 and CCL21. Our findings that LXR pathways and CCR7 are concomitantly upregulated in CAC_iv_ biology, could hint a possible role for CAC in reverse plaque remodeling.

We also demonstrated upregulation of FXR/RXR cholesterol-sensing nuclear receptors in the CAC_iv_ profile. Activation of FXR pathways in mouse models of atherosclerosis could almost completely inhibit aortic atherosclerotic lesion formation [32], attenuated the pro-inflammatory expression of IL1*ß*, IL6 and TNF [33] and negatively modulated NF*κ*B-mediated inflammation [34]. Furthermore, upregulation of the ABCA1 cholesterol transporter by FXR agonists in ApoE-deficient macrophages, led to the induction of an anti-atherogenic cholesterol ‘unloading' mechanism *in vivo* [33].

Finally, IPA analysis indicated riboflavin metabolism as significantly upregulated in CAC_iv_ biology. Riboflavin or vitamin B2 is the central element of the cofactors flavin mononucleotide (FMN) and flavin adenine dinucleotide (FAD). These cofactors are essential for mitochondrial oxidoreductase reactions, cellular oxidative stress resistance and endothelial nitric oxide synthase (eNOS) coupling and eNOS activity [35].

Together, the biological pathways observed in our CAC_iv_ profile, seem to relate to reverse cholesterol transport, immunomodulation, energy metabolism, oxidative stress resistance and NO bioavailability. These findings hint at a possible beneficial effect of CAC_iv_ therapy in the treatment of atherosclerotic and cardiovascular disease. Alternatively, pathophysiological conditions leading to impaired *in vivo* CAC function might induce endothelial dysfunction and atherosclerotic plaque formation and progression. However, future experimental studies are required to confirm these hypotheses.

In conclusion, our data indicate that CAC_iv_ are genotypically related to regulatory M2c macrophages. CAC_iv_, however, show little evidence of endothelial cell transdifferentiation. We propose new mechanisms by which CAC_iv_ could be efficiently applied in the broad field of cardiovascular pathophysiology, more specifically by immunomodulation, tissue remodeling, enhancement of cholesterol efflux and vasculoprotection.

## Data Availability

The microarray data used to support the findings of this study have been deposited and are publicly available in the Gene Expression Omnibus (GEO) repository (GSE2040 and GSE5099).

## Supplementary Materials

Supplementary Materials Basic statistical analysis of microarray data integrity.
