# Supplementary material for: Identification of Macrophage Genotype and Key Biological Pathways in Circulating Angiogenic Cell Transcriptome
Source: Stem Cells Int. 2019 May 2;2019:9545261. doi: 10.1155/2019/9545261 (PMC6525806; doi:10.1155/2019/9545261)

Supplementary data file

GSE 2040 microarray dataset

Experiment: Human endothelial progenitor cells (EPC) vs. human umbilical vein endothelial cells (HUVEC) vs. CD14+ monocytes

Data used in the present article:
3 samples of EPC
3 samples of CD 14+ monocytes
The HUCEX data was omitted.

Microarray characteristics:
HG-U95Av2 microarray (Affymetrix Inc.)
Targets 9,670 human genes as selected from the National Center for Biotechnology Information (NCBI) Gene Bank database with a total of 12,000 oligonucleotide sets.

Normalization method: Raw expression data were normalized using GCRMA

Box-and-whisker plot

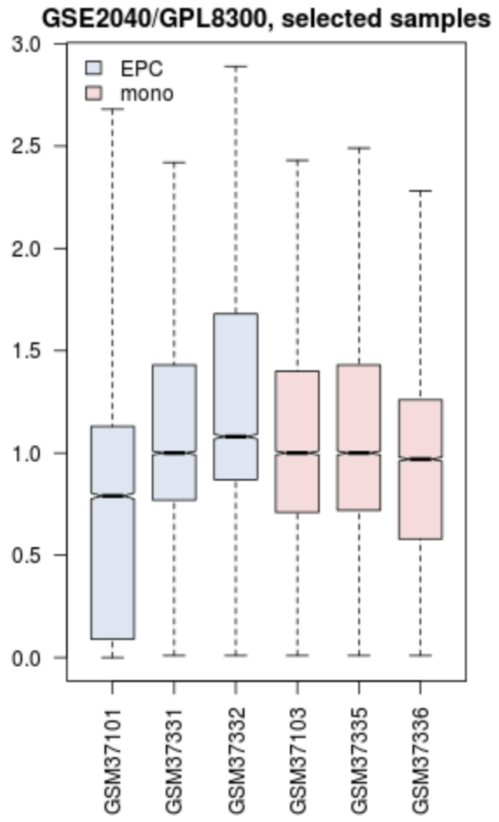

Volcano plot

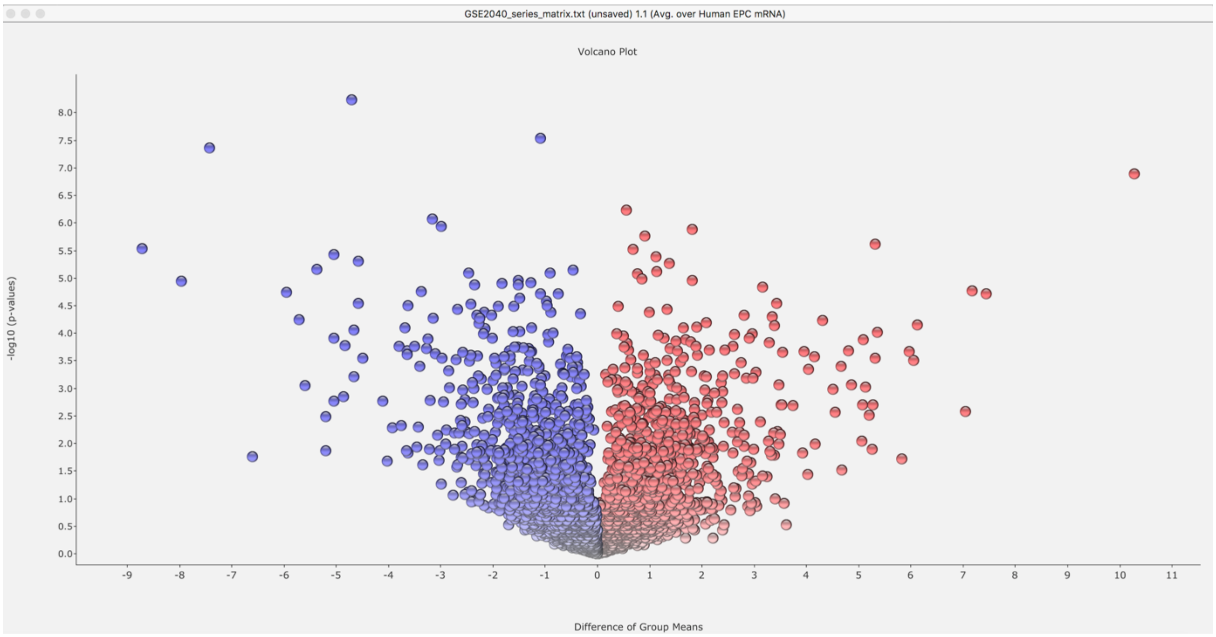

GSE 5099 microarray dataset

Experiment: Expression Data from Macrophage Maturation and Polarization Experiment

Cell type: Freshly isolated human monocytes were cultured in the presence of M-CSF (100 ng/ml) for 7 days, and then activated. The study includes Monocytes at day 0, macrophages at day 3 and 7, Interferon Gamma and LPS treated macrophages and IL-4 treated macrophages.

Microarray characteristics:
HG-U133: Affymetrix Human Genome U133 Array, 2 arrays with a total of 44928 entries.

Normalization method: Raw expression data were normalized using GCRMA

Box-and-whisker plot

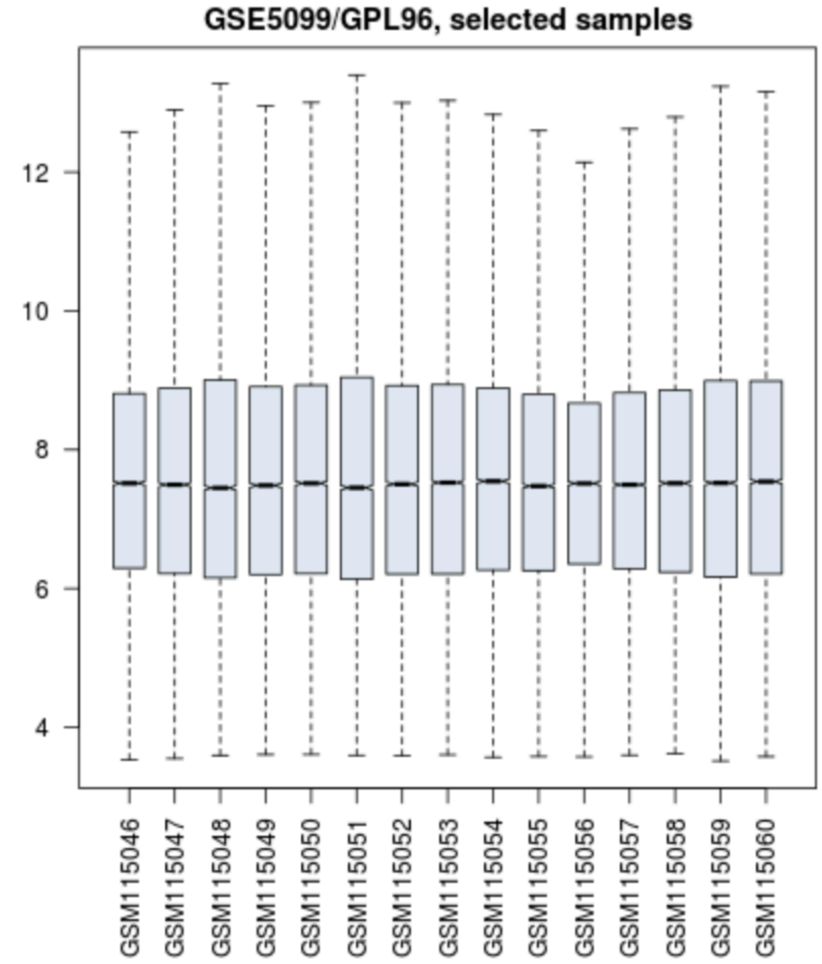

Volcano plot

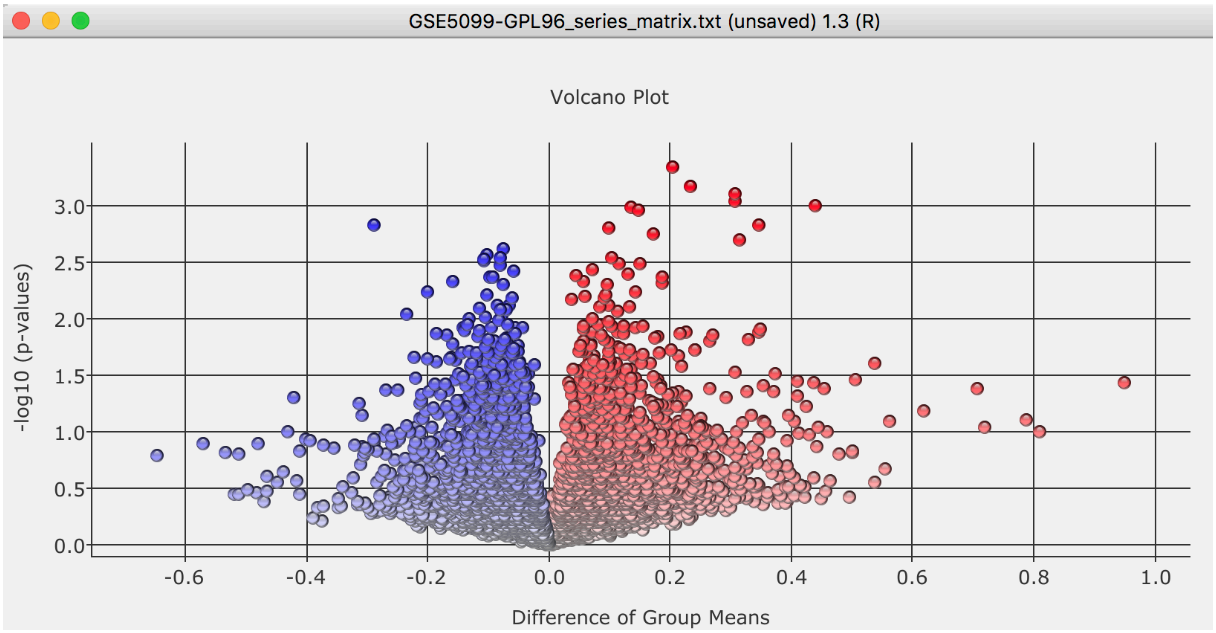

Supplement: Supplementary Materials — Basic statistical analysis of microarray data integrity. [file 9545261.f1.pdf]
